# Knowledge, Attitudes, Impact, and Anxiety Regarding COVID-19 Infection Among the Public in China

**DOI:** 10.3389/fpubh.2020.00236

**Published:** 2020-05-27

**Authors:** Yulan Lin, Zhijian Hu, Haridah Alias, Li Ping Wong

**Affiliations:** ^1^Fujian Provincial Key Laboratory of Environment Factors and Cancer, Department of Epidemiology and Health Statistics, School of Public Health, Fujian Medical University, Fuzhou, China; ^2^Centre for Epidemiology and Evidence-Based Practice, Department of Social and Preventive Medicine, Faculty of Medicine, University of Malaya, Kuala Lumpur, Malaysia

**Keywords:** COVID-19, knowledge, attitude, impact, anxiety

## Abstract

**Objectives:** Sufficient knowledge and positive attitudes are crucial to the prevention of COVID-19. However, little is known about public awareness and attitudes regarding COVID-19 in China. The impact of COVID-19 on the societal well-being and anxiety levels of the public has never been documented. The aim of this study was to survey the knowledge, attitudes, impact, and anxiety levels of the people of China in relation to the COVID-19 outbreak.

**Method:** A cross-sectional population survey using an online questionnaire was undertaken between Jan 24 and Feb 24, 2020. The study participants were residents of mainland China over the age of 18 years. The attitude items in this study measured the perceived threat of COVID-19 based on the Health Belief Model. Anxiety was measured with the State-Trait Anxiety Inventory (STAI), a self-reported questionnaire that measure both state (STAI-S), and trait anxiety (STAI-T)

**Results:** A total of 2,446 completed responses were received. The mean and standard deviation (SD) for the total knowledge score was 20.3 (*SD* ± 2.9) out of a possible score of 23. The social disruption and household economic impact were notable, particularly in provinces with higher cumulative confirmed cases. The majority of responses indicated a low perceived susceptibility of being infected (86.7% [95%CI 85.4–88.1]), with a fair proportion of respondents perceiving a higher severity (62.9% [95% CI 61.0–64.8]). The mean total impact score was 9.9 (*SD* ± 3.8) out of a possible score of 15. The mean score for STAI-S was 48.7 (*SD* ± 10.8), whereas the mean STAI-T score was 45.7 (*SD* ± 8.5). By demographics, women reported significantly higher odds for higher levels of both STAI-S (*OR* = 1.67) and STAI-T (*OR* = 1.30) compared to men. People of a younger age were also more likely to experience higher STAI-S and STAI-T. Higher perceived susceptibility and severity and impact were strong predictors of higher levels of STAI-S and STAI-T.

**Conclusion:** Our findings can assist in tailoring public communication to change people's knowledge and attitudes. The present study also underlined the importance of the promotion of mental health during infectious disease outbreaks to help in moderating the perceived threat, social and household economic impact, targeting the vulnerable segment of the population.

## Introduction

Starting on December 8, 2019, clusters of pneumonia cases were reported in Wuhan, Hubei province, China (1, 2). Subsequently, on January 7, 2020 the Chinese Centre for Disease Control and Prevention (CDC) identified a novel coronavirus linked to the outbreak, which was temporarily named 2019-novel coronavirus (2019-nCoV) by the World Health Organization (WHO) (3, 4). On February 11, 2020, the WHO declared the official name for the new coronavirus disease as Coronavirus disease 2019 (COVID-19). Despite considerable efforts to reduce transmission having been carried out, as of March 8, 2020, 3 months after the onset of the outbreak, a total of over 80 thousand laboratory-confirmed cases have been reported with deaths exceeding 3,000 (4). To date, concern is still mounting over the continuing spread of COVID-19 in China despite a decrease in confirmed cases having recently been observed.

In terms of knowledge and behavior, evidence has shown that disease-related literacy and attitudes of people in society play major roles in shaping their practices and controlling the spread of disease during an outbreak (5). Numerous empirical studies have also showed that health education can improve knowledge and change unfavorable attitudes and behaviors, effectively curbing infectious diseases and epidemics (6). To date, the knowledge and beliefs of the general public in China regarding COVID-19 is limited. Assessing COVID-19 related knowledge and health beliefs of the general public during the disease outbreak is critically important and would provide better insight to address knowledge and belief gaps relating to the disease, thus helping in the control and management of the current ongoing outbreak.

Outbreaks of emerging infectious diseases can be associated with considerable anxiety and fear in the general public or in specific communities, especially when the infection rate and deaths are substantial. During infectious disease outbreaks, tension surges in the entire community and results in a significant social and household economic effect (7). During the severe acute respiratory syndrome (SARS) and avian influenza outbreaks in China, travel, tourism, jobs, and retail businesses were reported to be greatly impacted (8). The mental health of members of the public directly affected by the COVID-19 epidemic has been under-addressed (9). During the current COVID-19 outbreak, tackling psychological and mental health problems of the lay public is crucial. Establishing normal anxiety responses, and mitigating fear and discrimination directed toward persons infected with, and affected by, infectious disease can be important in controlling transmission (10). Hence, investigation of the mental health consequences could provide information to the health authorities and help to provide mental health interventions to those who are in need. To date, the impact of COVID-19 on the societal well-being of the lay public in mainland China has never been reported. Little has also been reported regarding the psychological outcome during the current COVID-19 outbreak.

In this context, understanding the general publics' behavioral and psychological outcomes could provide useful information to policy makers, enabling the planning of educational interventions on the population to effectively curb the outbreak. Therefore, to address the gap in the literature, the primary objective of the present investigation was to examine the level of knowledge, attitudes, impact, and anxiety levels of people in mainland China during the ongoing COVID-19 outbreak.

## Methods

### Study Design and Participants

We commenced a 1 month (Jan 24 to Feb 24, 2020) cross-sectional, web-based anonymous survey using an online questionnaire. The survey link was advertised in WeChat, the most popular and widely used social media platform in China. The inclusion criteria were that the respondents were mainland China residents who were between 18 and 70 years of age. Upon completing the survey, the participants were encouraged to disseminate the survey link to all their contacts with a thank you note at the end. The participants were informed that their participation was voluntary, and consent was implied through their completion of the questionnaire.

The questionnaire was developed in English and was then translated into Chinese. Local experts validated the content of the questionnaire, after which it was pilot tested. The survey consisted of three sections, which assessed (1) demographic background, (2) knowledge about COVID-19, (3) attitudes, (4) impact, and (5) anxiety symptoms.

#### Knowledge

The participants' knowledge was assessed using a series of questions regarding signs and symptoms of COVID-19 infection, transmission, and protection from infection (23 item scale). The response options were “true,” “false,” or “don't know.” A correct response was given a score of one and an incorrect or “don't know” response was scored zero. The possible total knowledge score ranged from 0 to 23, with higher scores representing higher levels of knowledge.

#### Attitudes

In the Health Belief Model, threat perceptions depend upon the perceived susceptibility to the illness and the perceived severity of the consequences of the illness (11). The attitudes items in this study measured the perceived threat of COVID-19, using a combination of questions on perceived susceptibility and perceived severity. The perceived susceptibility questions measured an individual's belief in the likelihood of being infected with COVID-19 and the perceived severity questions measured the consequence of a COVID-19 infection. The response options were “extremely,” “very,” “moderate,” and “not at all.”

#### Impact

A five-item question was developed to measure the social disruption and household economic impact of COVID-19. Participants were asked to rate how COVID-19 had affected five functions (non-work related travel, work related travel, family's daily routine, job, and finance) using a four-point scale (0 = not at all, 1 = very little, 2 = somewhat, 3 = a great extent). The possible total impact score ranged from 0 to 15, with higher scores representing higher levels of impact.

#### Anxiety Symptoms

Anxiety was measured by means of the State-Trait Anxiety Inventory (STAI), a 40-item self-reported questionnaire that measure both state (STAI-S) and trait anxiety (STAI-T) (12). The STAI is the gold standard for measuring anxiety and stress (13) with the STAI-S subscale measuring the anxiety at the moment of scoring. State anxiety is conceptualized as a transient emotional condition of the individual, characterized by subjectively experienced feelings of tension, together with a heightened activity of the autonomous nervous system. The STAI-T refers to anxiety proneness; that is, relatively stable individual differences in the tendency to react with a more intense state anxiety in situations that are perceived as threatening (14). The items are summed per scale and transformed into scores between 20 and 80. Both STAI-S and STAI-T consist of 20 items, each with four point Likert scales. Scores thus range between 20, indicating a low level of anxiety and 80, indicating a high level. A score of 40 or higher has been reported to reflect anxiety symptoms (15, 16).

### Ethical Considerations

This study protocol was approved by the Research Ethics Committee of the Fujian Medical University.

### Statistical Analyses

The reliability of the knowledge and impact scores was evaluated by assessing the internal consistency of the items representing the scores. The 23 items for knowledge and the five items impact scores had a reliability (Cronbach's α) of 0.803 and 0.852, respectively. The reliability computed for STAI-S and STAI-T was found to be 0.777 and 0.827, respectively.

Multivariable logistic regression, using a simultaneous forced-entry method, was used to determine the factors influencing high anxiety scores for both the STAI-S and STAI-T subscales. Odds ratios (OR), 95% confidence intervals (95%CI), and *p*-values were calculated for each independent variable. The model fit was assessed using the Hosmer–Lemeshow goodness-of-fit test (17). All statistical analyses were performed using the Statistical Package for the Social Sciences, version 20.0 (IBM Corp., Armonk, NY, USA). The level of significance was set at *p* < 0.05.

## Results

A total of 2,446 complete responses were received in the month's survey. As shown in Table 1, the majority of the respondents were female (70.0%) and of age 18–24 years (68.4%). The majority of the respondents were from provinces where the number of confirmed COVID-19 cases was 1,000–10,000 (83.8%). Only 84 (3.4%) were from the provinces with above 10,000 confirmed cases, namely the province of Hubei and Hunan, the epicenter of COVID-19.

**Table 1:** Demographic characteristics of respondents (*N* = 2,446).

	***N* (%)**
**Socio demography**	
**Age group (years)**	
18–24	1,674 (68.4)
25–39	537 (22.0)
40–68	235 (9.6)
**Gender**	
Male	733 (30.0)
Female	1,713 (70.0)
**Highest educational level**	
High school and below	367 (15.0)
University	2,079 (85.0)
**Annual average household income (RMB)**	
<50,000	838 (34.3)
50,000–120,000	944 (38.6)
>120,000	664 (27.1)
**Locality**	
Urban	1,451 (59.3)
Suburban/ Rural	995 (40.7)
**Provinces by cumulative number of confirmed cases**^†^	
<500	61 (2.5)
500–999	251 (10.3)
1,000–10,000	2,050 (83.8)
>10,000	84 (3.4)

† List of provinces under cumulative number of confirmed cases as of February 24, 2020. >10,000 (Hubei, Hunan).

1,000-10,000 (Guangdong, Sichuan, Zhejiang, Shandong, Heilongjiang, Chongqing, Beijing).

500–599 (Hainan, Jiangsu, Henan, Fujian, Hebei, Anhui, Jiangxi, Guizhou, Shanxi, Liaoning, Shanghai, Tianjin, Guangxi).

*<500 (Yunnan, Shaanxi, Jilin, Gansu, Inner Mongolia, Xinjiang, Ningxia, Tibet)*.

### Knowledge and Attitudes

**Figure 1:**
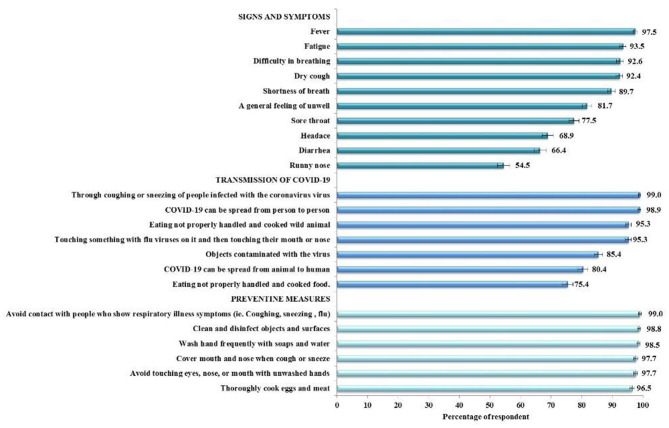
Percentages of correct responses to knowledge items (*N* = 2,446).

Figure 1 shows the proportion of correct responses to knowledge items. The highest proportion of correct responses for transmission and protection measures against COVID-19 were found. A knowledge gap in the signs and symptoms of COVID-19 infections, namely runny nose (54.5%), diarrhea (66.4%), headache (68.9%), and sore throat (77.5%) was found. The mean and standard deviation (*SD*) for the total knowledge score was 20.3 (*SD* ± 2.9) out of a possible score of 23. The median was 21 (inter quartile range, IQR, 19–23). The knowledge scores were categorized as a score of 21–23 or 0–20 based on the median split; as such, a total of 1,367 (55.9%; 95%CI 53.9–57.9) were categorized as having a score of 21–23 and 1,079 (44.1%; 95%CI 42.1–46.1) had a score of 0–20.

The results of attitudes showed that the majority (*n* = 2,121, 86.7% [95%CI 85.4–88.1]) reported “moderately likely/not at all” of being infected with COVID-19. Nonetheless, a smaller proportion (*n* = 1,538, 62.9% [95% CI 61.0–64.8] reported an “extremely likely/very likely” worry of the consequences of getting COVID-19.

### Impact

**Figure 2:**
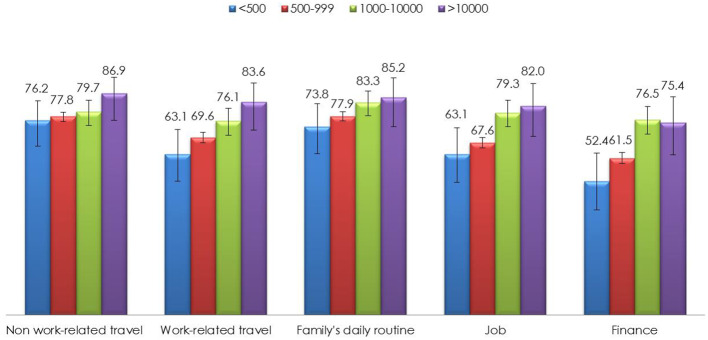
Proportion of “A great extent/somewhat” responses for impact of COVID-19 items by provinces with cumulative confirmed cases (*N* = 2,446).

Figure 2 summarizes the findings of the impact items according to provinces by cumulative confirmed cases. In general, participants from provinces with a higher number of cumulative confirmed cases reported a higher proportion of “a great extent/somewhat” response for all the impact items. The mean and *SD* for the total impact score was 9.9 (*SD* ± 3.8) out of a possible score of 15. The median was 10 (IQR, 7–13). The impact scores were categorized as a score of 10–15 or 0–9 based on the median split; as such, a total of 1,424 (58.2%; 95%CI 56.2–60.2) were categorized as having a score of 10–15 and 1,022 (41.8%; 95%CI 39.8–43.8) had a score of 0–9.

### Anxiety Symptoms

The mean score (*SD*) for STAI-S was 48.7 (10.8), whereas it was 45.7 (8.5) for STAI-T. Among the study respondents, a total of 1,915 (78.3%; 95%CI 76.6–79.9) showed high levels (a score of 40 or higher) of STAI-S and 1,869 (76.7%; 95%CI 74.9–78.3) showed high levels of STAI-T. Educational level, classification of provinces with number of confirmed COVID-19 cases and knowledge scores were not significantly associated with either STAI-S or STAI-T in the univariate analyses. Table 2 shows the multivariable analyses of factors associated with high STAI-S and STAI-T. The younger age group respondents, females, and those residing in suburban/rural areas were more likely to express high levels of STAI-S. Perceived susceptibility and severity were the two strongest predictors of higher levels of STAI-S. Having higher impact score was also significantly associated with high STAI-S. Multivariable logistic analyses for factors associated with high STAI-T showed that younger age respondents and females were more likely to have high levels of state STAI-T. Lower income was a significant predictor of high STAI-T, although not for STAI-S. Similar to STAI-S, higher perceived susceptibility and severity and high impact scores were also strong predictors of higher levels of STAI-T.

**Table 2:** Multivariable logistic analyses of factors associated with total State-Trait Anxiety Score (*N* = 2,446).

**Covariates**	**State Anxiety Score (STAI-S)^†^**	**Trait Anxiety Score (STAI-T)^‡^**
	**41–80 vs. 20–40**	**41–80 vs. 20–40**
	**OR (95%CI)**	**OR (95%CI)**
**SOCIO DEMOGRAPHY**		
**Age group (years)**		
18–24	1.70 (1.23–2.36)^**^	3.01 (2.21–4.10)^***^
25–39	1.59 (1.11–2.29)^*^	1.79 (1.28–2.51)^**^
40–68	Ref	Ref
**Gender**		
Male	Ref	Ref
Female	1.67 (1.35–2.05)^***^	1.30 (1.05–1.61)^*^
**Annual average household income (RMB)**		
<50,000	1.30 (0.98–1.71)	1.70 (1.30–2.23)^***^
50,000–120,000	1.14 (0.89–1.47)	1.42 (1.12–1.81)^**^
>120,000	Ref	Ref
**Locality**		
Urban	Ref	Ref
Suburban/ Rural	1.35 (1.08–1.69)^**^	1.13 (0.91–1.41)
**PERCEIVED SUSCEPTIBILITY**		
**Likelihood to be infected**		
Moderate likely/Not at all	Ref	Ref
Extremely/Very likely	2.21 (1.49–3.27)^***^	1.61 (1.15–2.27)^**^
**PERCEIVED SEVERITY**		
**Worry of consequences**		
Moderate likely/Not at all	Ref	Ref
Extremely/Very likely	2.74 (2.23–3.37)^***^	2.26 (1.85–2.77)^***^
**IMPACT**		
**Total impact score**		
Score 0–9	Ref	Ref
Score 10–15	1.79 (1.46–2.20)^***^	1.78 (1.46–2.18)^***^

* p < 0.05,

** p < 0.01,

*** p < 0.001.

† Hosmer–Lemeshow test, chi-square: 3.972, p-value: 0.860; Nagelkerke R^2^: 0.145.

‡ *Hosmer–Lemeshow test, chi-square: 1.042, p-value: 0.199; Nagelkerke R^2^: 0.142*.

## Discussion

To our knowledge, ours is the first investigation aimed to associate the knowledge, attitudes, and psychological response to COVID-19 during the ongoing outbreak, and the findings could offer an insight into the strategies required to effectively address these important public health issue.

In general, the study participants have a good level of COVID-19 related knowledge. The need to improve knowledge of the signs and symptoms of the infection are clearly shown from the results of this study. In particular, findings indicate that a considerable proportion of surveyed participants lack knowledge of important symptoms such as runny nose and diarrhea. Also of particular concern is the relatively few study participants who are aware that eating food that had not been properly handled and cooked could also be a source of transmission.

It is important to note that a large majority of the participants in this study expressed a low level of susceptibility in contracting COVID-19. Nonetheless, a relatively higher proportion expressed a high perceived severity of COVID-19. Latest evidence showing the case fatality rate (CFR) of 2.3% in COVID-19 has been reported (18). For comparison, Middle East respiratory syndrome (MERS) and SARS exhibited CFRs of 35 and 15%, respectively (19, 20). Further, mortality among COVID-19 victims appears highest for the elderly and those with multiple comorbidities (21). It is widely reported in news media that, in comparison to SARS and MERS coronaviruses, COVID-19 is probably more highly transmissible but not as deadly. Despite epidemiological data showing high transmissibility of COVID-19, high proportion reported low susceptibility of contracting the virus. Reason for perceived susceptibility is not explored in this study and warrant further investigation. Of a positive note, a fair proportion of respondents perceiving a higher severity of the COVID-19 infection.

Results from this study also indicated that a sizable proportion of study participants were greatly impacted in non-work related travel, work related travel, family's daily routine, job, and finance by the COVID-19 outbreak. Although official data on the economic impact of COVID-19 are not yet available, the high impact on work-related travel, job, and finance reported by our study participants indicated the potential catastrophic impact of COVID-19 to the economics of China. The increase in impact is in line with the increase in the provinces with higher numbers of cumulative confirmed cases, implying that the intensity of impact is higher in areas with more confirmed cases. As such, recovery initiatives to minimize the impact of COVID-19 should focus on areas with the highest cumulative confirmed cases.

The study participants have a high level of anxiety, with nearly 80% reporting a score above 40 in both the STAI-S and STAI-T subscales. In particular, the mean score for both STAI-S and STAI-T in this study was 48.7 (*SD* ± 10.8) and 45.7 (*SD* ± 8.5), respectively, which is similar to those reported among doctors (47.8 [*SD* ± 11.1]), hospital administrative staff (47.1 [*SD* ± 10.6]), and allied health workers (47.8 [*SD* ± 10.9]) in Hong Kong during the SARS outbreak in 2003 (22). The similarity to the anxiety levels of frontline health workers during SARS implies high levels of anxiety among the lay public during the current COVID-19 outbreak. Infectious disease outbreaks are frequently characterized by social disruption and an overall climate of fear, and in the COVID-19 outbreak, daily reporting of the exponential increase in confirmed cases and death rates over the news media could be the leading cause of high anxiety levels. Our findings support the crucial need for the development and implementation of mental health assessment, support, treatment, and services for the public in mainland China due to the COVID-19 outbreak (9). It is particularly important for the public to maintain a moderate level of anxiety as extreme anxiety may impair immune system functioning and increase the risk of infection (23).

By demographics, gender differences in anxiety response to COVID-19 were observed in this study. Women reported significantly higher odds of high levels of both STAI-S (*OR* = 1.67) and STAI-T (*OR* = 1.30) compared to men. People of a younger age are also more likely to experience high anxiety levels. The reason why the younger age group respondents expressed a higher degree of anxiety was not investigated in this study, and thus warrants further investigation. The use of social media is much more popular among younger people. The huge amount of information about the novel coronavirus circulated on social media perhaps spark anxiety among the younger people.

The identified demographic disparities in anxiety levels provide insights for targeted interventions. It is suggested that public health interventions should be carried out to reach the identified groups in order to reduce the mental-health-related burden. In this study, the level of knowledge of COVID-19 does not have any significant influence on anxiety levels. Nonetheless, having a higher perception of susceptibility and severity were the most important predictors of high levels of STAI-S and STAI-T. Previous studies have reported the benefit of a higher perception of susceptibility and severity in triggering a higher practice of recommended prevention measures during disease outbreak (24–26). Therefore, it is important to maintain a moderate amount of perceived susceptibility and severity of disease to promote prevention practices, as overtly high level may adversely result in high anxiety distress among the population.

Another clear lesson learned from SARS is that the effect of an infectious disease outbreak alters the economic and social patterns of people's lives, which negatively influences the psychological well-being of the people (26). Likewise, this study found the increased effect of social and household economic impact associated with COVID-29 was another significant predictor of high anxiety levels. The COVID-19 outbreak prompted nationwide localized school closures, restricted travel, a sharp decrease in business activity, and threatened job security for many people in China. Thus, there is a crucial need to ensure that mental health intervention focuses on the segment of the population that face serious social disruption and household economic impact

As with all studies, it is worth noting a few limitations of the present study, particularly with regard to the study design and data collection method. Firstly, due to the cross-sectional methodology, the directionality of the association or the causal relationship between the knowledge, health beliefs, and anxiety levels could not be established; however, the findings provide a basis for acquiring and testing a causal hypothesis. Due to various resource limitations in the midst of the disease crisis in China, using an online web-based questionnaire via a social media platform may lead to selection bias, as reflected in the large sample of young adults and relatively small sample of respondents from the epicenter. Despite the lack of representativeness, this study's collected sample is of diverse demographics in which the findings provide valuable information for targeted health disparity intervention. In spite of these limitations, this is the first survey of its kind from China, the country where the virus is believed to have originated and recorded the largest number of infections. The current study provides useful first-hand information of the knowledge, attitudes, anxiety levels, and influencing factors among the public in China.

## Conclusion

The results of the current study showed that the public in China had a considerably good level of knowledge related to COVID-19, with the need to increase knowledge about disease signs and symptoms. The high levels of anxiety found in this study warrant the implementation of effective preventive and emotional regulation control strategies. The present study underlines the importance of the promotion of mental health during infectious disease outbreaks, targeted at the vulnerable segment of the population, to moderate perceived threats, social, and household economic impact.

To date, as of 2nd May, 2020, China has successfully flattened the curve of the COVID-19 infection. Many other countries in the world are still fighting the spread of the new coronavirus. Our findings provide valuable insights for countries that are still facing the ongoing pandemic to identify and address gaps in knowledge and detrimental attitudes, as well as addressing the mental health impact of COVID-19.

## Data Availability Statement

The raw data supporting the conclusions of this article will be made available by the authors, without undue reservations, to any qualified researchers.

## Ethics Statement

The studies involving human participants were reviewed and approved by Research Ethics Committee of the Fujian Medical University. The patients/participants provided their written informed consent to participate in this study.

## Author Contributions

LW and YL conceived the study. YL collected data. LW and HA analyzed the data. LW wrote the manuscript. All authors have approved the manuscript.

## Conflict of Interest

The authors declare that the research was conducted in the absence of any commercial or financial relationships that could be construed as a potential conflict of interest.
